# A study on the effects of exercise training on cortical excitability in athletes: a meta-analysis based on TMS measurements

**DOI:** 10.3389/fpsyg.2025.1627227

**Published:** 2025-09-19

**Authors:** Xinhua Jiang, Lei Li, Yang Liu, Weiwei Feng

**Affiliations:** ^1^Department of Physical Education, Guangdong Polytechnic of Science and Trade, Guangzhou, China; ^2^School of Physical Education, Nanchang Normal University, Nanchang, China; ^3^School of Physical Education, Guangzhou Huashang College, Guangzhou, China

**Keywords:** exercise training, cortical excitability, athletes, meta-analysis, transcranial magnetic stimulation (TMS), neuroplasticity, motor cortex, athletic performance

## Abstract

**Objective:**

This study investigates the impact of exercise training on athletes’ cortical excitability, aiming to provide scientific evidence for optimizing training protocols and enhancing athletic performance.

**Method:**

Following PRISMA guidelines, a systematic search was conducted in databases including PubMed, Web of Science, Embase, and Cochrane Library up to May 1, 2025, including randomized controlled trials *(RCTs)* or quasi-experimental studies using transcranial magnetic stimulation *(TMS)* to assess cortical excitability in athletes. Study quality was evaluated using the Cochrane Risk of Bias tool. Meta-analysis was performed with RevMan 5.4 software, using standardized mean difference *(SMD)* as the effect size and a random-effects model to analyze heterogeneity. Sensitivity analysis was conducted using Stata 18.0.

**Results:**

The meta-analysis included 8 studies (245 participants). Results showed that exercise training significantly enhanced cortical excitability (*n* = 8, SMD = −1.2, 95% CI = −1.75 to −1.65, *p* < 0.01), with high heterogeneity (*I*^2^ = 71%). Subgroup analysis by exercise type indicated significant effects for combat sports and endurance sports (*p* < 0.05), but not for technical-tactical sports (*p* > 0.05). Subgroup analysis by training duration showed significant effects for long-, medium-, and short-term training (*p* < 0.05), with medium-term training exhibiting low heterogeneity (*I*^2^ = 0%). Sensitivity analysis and funnel plots confirmed robust results with low risk of publication bias.

**Conclusion:**

Exercise training significantly enhances athletes’ cortical excitability, particularly in combat and endurance sports and during medium-term training. Future research should further explore the specific effects of different training types and TMS metrics to reduce heterogeneity and optimize training design.

**Systematic review registration:**

The systematic review has been registered in PROSPERO under the ID CRD420251045271. The registration details are available at: https://www.crd.york.ac.uk/PROSPERO/recorddashboard.

## 1 Introduction

Cortical excitability, defined as the responsiveness of cortical neurons to stimuli, is pivotal for understanding neural adaptations to exercise training. This responsiveness reflects the ease with which neurons generate action potentials, modulated by the balance between excitatory neurotransmitters and inhibitory neurotransmitters (Grall-Bronnec and Sauvaget, 2014; Ziemann et al., 2015). In 1985, Barker and colleagues introduced transcranial magnetic stimulation (TMS) at the 11th World Congress of Clinical Neurophysiology and Electroencephalography in London (Barker and Jalinous, 1985). This non-invasive technique employs magnetic fields to stimulate the primary motor cortex, eliciting motor-evoked potentials (MEPs) in contralateral muscles, revolutionizing the study of cortical excitability. TMS operates on Faraday’s principle of electromagnetic induction, whereby a transient current in a coil generates an induced magnetic field. This rapidly changing magnetic field penetrates the skull and soft tissues, inducing an electric current in intracranial conductors opposite to the coil’s current direction. This current acts on localized cortical regions, facilitating synaptic transmission to adjacent neurons, altering the polarization state near cortical neurons, and modifying neuronal membrane potentials to trigger a cascade of physiological and biochemical responses (Barker, 1991; Bütefisch et al., 2004; Cao et al., 2011; Carroll et al., 2001; Fadiga et al., 1995). Characterized by its non-invasive, painless nature, unique functionality, operational simplicity, and high reliability (Lioumis et al., 2025; Moscatelli et al., 2021) TMS has emerged as a highly promising clinical tool. It is widely applied in the treatment of neurological and psychiatric disorders, including anxiety (Wang et al., 2023) depression (Akpınar et al., 2022; Delaney et al., 2025), obsessive-compulsive disorder (Carmi et al., 2019), Parkinson’s disease (Chung et al., 2020), and Alzheimer’s disease (Sabbagh et al., 2020).

In recent years, the application of TMS in sports science has markedly expanded (Kallioniemi et al., 2025) with extensive use in investigating post-exercise central fatigue, sensorimotor integration, motor coordination, and neural plasticity (Höllge et al., 1997a; Matsumoto et al., 2013). For instance, TMS studies have demonstrated that maximal voluntary contraction (MVC) enhances corticospinal excitability (Touge et al., 2012). Similarly, several weeks of skill training can augment corticospinal pathway excitability (Jensen et al., 2005). Moscatelli et al. further elucidated that exercise training influences both central and peripheral nervous systems, resulting in heightened cortical excitability among athletes, characterized by faster neural signal transmission and reduced reaction times (Moscatelli et al., 2016b). These findings underscore the potential of TMS in elucidating how training-induced neural adaptations optimize athletic performance.

Cortical excitability is quantitatively assessed through TMS-derived metrics, including resting motor threshold (rMT), motor-evoked potential (MEP) amplitude, MEP latency, short-interval intracortical inhibition (SICI), and intracortical facilitation (ICF). The rMT represents the minimal stimulation intensity required to elicit a muscle response, while MEP amplitude and latency reflect the strength and conduction velocity of corticospinal output, respectively (Wassermann, 1998). SICI, observed when a subthreshold conditioning stimulus precedes a suprathreshold test stimulus by 1–6 milliseconds, indicates GABA-mediated inhibitory processes (Thompson et al., 1991). In contrast, ICF, occurring at intervals of 8–30 milliseconds, reflects excitatory processes (Kiers et al., 1997). These metrics are critical for elucidating the balance between excitation and inhibition within the motor cortex, which is essential for motor control and learning (Bestmann and Krakauer, 2015). Although the precise mechanisms underlying ICF facilitation remain incompletely understood, they are likely associated with heightened cortical and spinal excitability (Ziemann, 1999).

Long-term systematic training is reported to induce neuroplastic changes in the nervous system, characterized by enhanced cortical excitability and improved corticospinal conduction efficiency, which are closely linked to athletic performance (Jensen et al., 2005; Moscatelli et al., 2020). These adaptive changes likely stem from repeated activation of motor circuits, strengthened synaptic connections, and alterations in neurotransmitter dynamics (Bestmann and Krakauer, 2015). For instance, animal studies suggest that exercise training enhances synaptic efficacy in the motor cortex through mechanisms akin to long-term potentiation (LTP) (Rioult-Pedotti et al., 2000). In humans, TMS studies reveal that athletes exhibit lower rMT and higher motor-evoked potential (MEP) amplitudes compared to non-athletes, indicative of heightened cortical excitability (Fulton et al., 2002; Moscatelli et al., 2016a; Moscatelli et al., 2016c). These adaptations are considered foundational to the faster reaction times, improved motor coordination, and enhanced movement precision observed in trained individuals (Kujirai et al., 1993).

The specific effects of different training tasks or sport types on athletes’ cortical excitability remain unclear, with variations in stimulation intensity and intervals further influencing excitability measurements. For instance, single-pulse TMS primarily assesses corticospinal output, whereas paired-pulse paradigms probe intracortical circuits (Kujirai et al., 1993). Additionally, participant-related factors, such as training history, sex, and fatigue status, modulate excitability outcomes. These complexities underscore the need for systematic analyses to reconcile conflicting research findings. Prior studies, such as those by Moscatelli et al., have highlighted TMS as a tool for investigating motor cortex excitability but have not quantified its effects (Moscatelli et al., 2021), while others have described simplified methods for measuring the complexity of MEP (Spampinato et al., 2023). A systematic review and meta-analysis by Cavaleri et al. examined the number of stimuli required to assess cortical excitability and primary motor cortex function using TMS but focused on healthy individuals rather than athletes (Cavaleri et al., 2017). Therefore, this study aims to systematically evaluate the overall effect of exercise training on athletes’ cortical excitability through a meta-analysis, with a particular focus on potential differences across sport types. Leveraging quantitative data from TMS, this research seeks to analyze sources of heterogeneity and provide a scientific basis for optimizing training protocols and advancing the understanding of neural plasticity mechanisms.

## 2 Research methods

### 2.1 Registration

This study adheres to the PRISMA guidelines to ensure comprehensive and transparent reporting of methods and results (Moher et al., 2009). The research protocol has been registered on the PROSPERO platform under the registration number CRD420251045271.

### 2.2 Literature search strategy

This study searched four databases—PubMed, Web of Science, Embase, and Cochrane Library—up to May 1, 2025. Additionally, reference lists of included studies were manually reviewed to identify further eligible studies. The detailed search strategy is provided in Appendix.

### 2.3 Inclusion and exclusion criteria

Included studies had to meet the following criteria: (1) Participants were athletes with systematic, structured training experience, without neurological disorders, sports injuries, or other major health issues, with no restrictions on gender or ethnicity; (2) Intervention or control conditions used transcranial magnetic stimulation (TMS) to measure cortical excitability indicators; (3) TMS-assessed cortical excitability was the primary outcome, including measures such as MEP, RMT, or MT; (4) For non-athlete controls, participants could be recreational exercisers or sedentary individuals but must have no history of neurological disorders; (5) Study designs included randomized controlled trials (RCTs), crossover trials, or prospective experimental studies.

Exclusion criteria were as follows: (1) studies involving non-athlete populations; (2) studies that did not use TMS to assess cortical excitability or failed to provide specific quantitative outcome metrics; (3) studies limited to qualitative analyses, theoretical reviews, or conference abstracts lacking original data; (4) studies with incomplete original data, precluding effect size calculation; (5) conference abstracts, book chapters, or brief articles in languages other than English or Chinese.

### 2.4 Data extraction and risk of bias assessment

Two researchers independently conducted literature screening and data extraction. Initial screening was performed based on titles and abstracts, with any discrepancies resolved through discussion in accordance with predefined inclusion criteria until consensus was reached. Full-text reviews of selected studies were then conducted, and reference lists were traced to identify additional relevant studies. Extracted data included: (1) study characteristics; (2) TMS parameters; (3) primary outcome measures and their assessment methods; (4) study design features.

Two researchers independently evaluated the risk of bias for each study based on the seven domains outlined in the Cochrane Handbook for Systematic Reviews. These domains included: random sequence generation (selection bias), allocation concealment (selection bias), blinding of participants and personnel (performance bias), blinding of outcome assessment (detection bias), incomplete outcome data (attrition bias), selective reporting (reporting bias), and other biases. In cases of disagreement, a third researcher was consulted to reach a consensus. The finalized study characteristics and bias assessments were imported into Review Manager 5.4 to generate risk of bias graphs.

### 2.5 Data analysis

In the meta-analysis, we selected the standardized mean difference (SMD) as the primary effect size, and all forest plots were generated using RevMan 5.4 software, which adopts Cochran’s *Q* as the default metric for continuous variables. To address potential bias from small sample sizes, we recalculated Hedges’s g using Stata 18.0. The results showed that the pooled effect size based on Hedges’s *g* was nearly identical to the SMD, indicating that small-sample corrections did not significantly impact the study conclusions. Therefore, to maintain consistency with the software output and figure formats, we retained SMD in the main analysis and figures. This approach aligns with the recommendations of the *Cochrane Handbook for Systematic Reviews of Interventions* (Cumpston et al., 2019), which states that SMD is an acceptable effect size for comparing continuous variables across different scales. Additionally, the *I*^2^ statistic, ranging from 0 to 100%, was used to assess the degree of heterogeneity among studies. A fixed-effects model was applied when *I*^2^ ≤ 50%, while a random-effects model was used when *I*^2^ > 50%, with subgroup analyses conducted to identify and explore potential sources of heterogeneity. Statistical significance was inferred when *p* < 0.05; otherwise, results were considered non-significant.

The meta-analysis showed high heterogeneity among included studies (*I*^2^ = 71%), indicating significant inconsistency in results. Meta-regression analysis using Stata examined training duration, participant age, and intervention type as moderating variables, all yielding *p* > 0.05, suggesting these were not sources of heterogeneity (details in the Supplementary File). To more accurately estimate the overall effect of exercise training on cortical excitability, a random-effects model was used for data synthesis (Barili et al., 2018). Given the high heterogeneity, subgroup analyses were conducted to explore potential sources, including athlete type and training duration. To verify result reliability, sensitivity analysis was performed using a fixed-effects model (Rendina-Gobioff, 2006), and sequential exclusion of individual studies showed no significant change in the pooled effect size, confirming strong result robustness and enhancing the credibility of the study conclusions.

## 3 Results

### 3.1 Literature screening results

**Figure 1:**
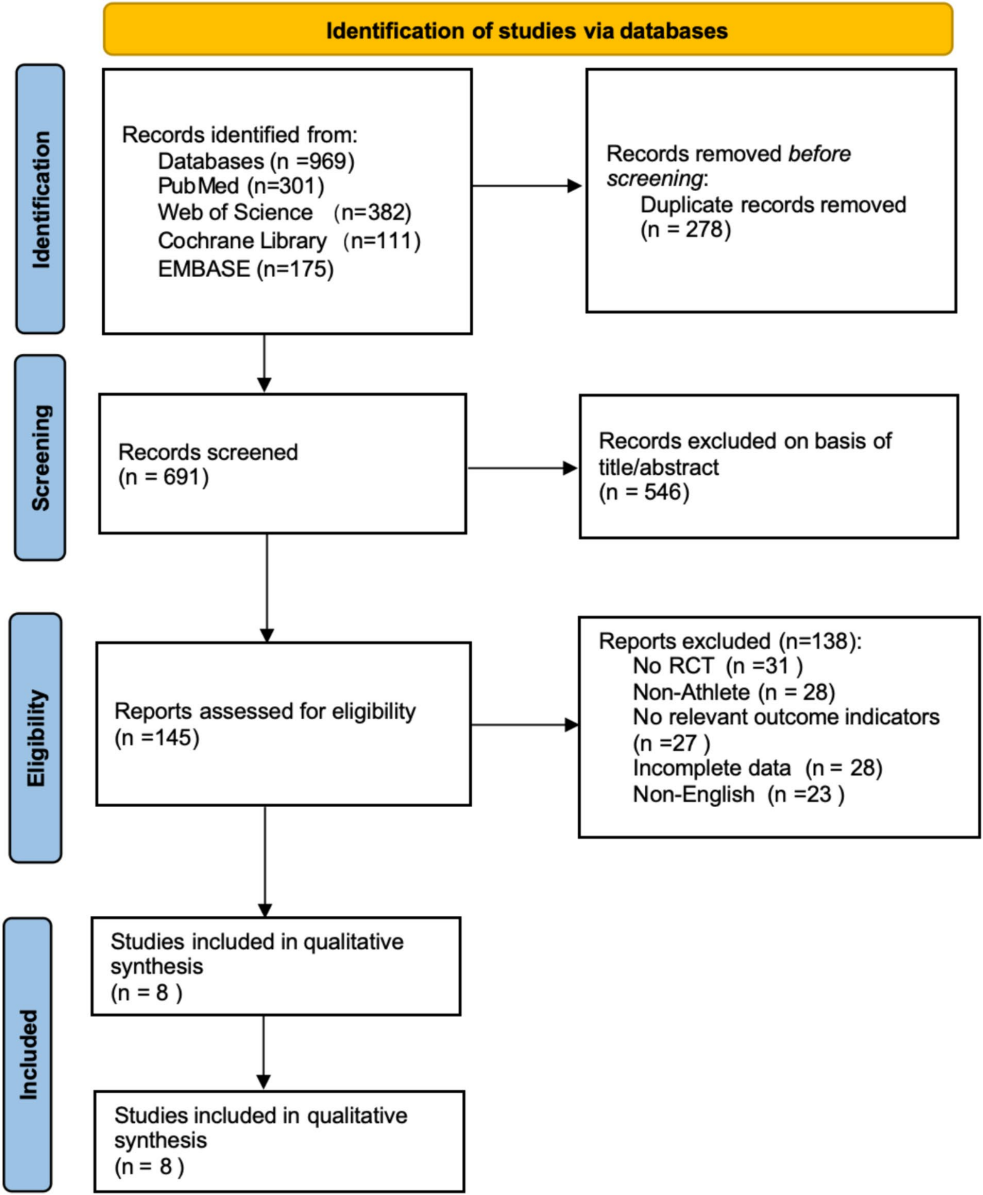
Literature screening flowchart (PRISMA declaration format).

Based on the search strategy, a total of 969 articles were initially retrieved (Web of Science: 382, PubMed: 301, EMBASE: 175, Cochrane Library: 111). All articles were imported into EndNote 21 for management, and 278 duplicates were removed, leaving 691 articles. After screening titles and abstracts, 546 articles irrelevant to the study topic or classified as reviews were excluded. Following abstract screening, 138 articles were excluded for the following reasons: non-RCT (*n* = 31), non-athlete participants (*n* = 28), no relevant outcome indicators (*n* = 27), incomplete data (*n* = 28), and non-English (*n* = 23). Ultimately, 8 articles were included in the meta-analysis. The literature screening process is shown in Figure 1.

### 3.2 Characteristics of included studies

The basic characteristics of the included studies are shown in Table 1. All studies were prospective, with 3 being randomized controlled trials (RCTs) and the rest being matched trials or cross-sectional comparisons. Participants were healthy, injury-free athletes aged 21–26 years. The studies spanned from 2002 to 2023, with most published in the last decade, reflecting growing interest in TMS in exercise training research. A total of 245 participants were included, approximately 70% male, covering various sports, including endurance events {rowing (Fulton et al., 2002), middle- and long-distance running (Perciavalle et al., 2010), combat sports [taekwondo (Moscatelli et al., 2016c), karate (Monda et al., 2017; Moscatelli et al., 2016a; Moscatelli et al., 2016b)]}, and technical-tactical sports (volleyball; Christiansen et al., 2017; Moscatelli et al., 2023). TMS stimulation primarily used single pulses, with figure-eight coils targeting the M1 and DLPFC. Common neurophysiological measures included MEP and RMT. Most studies found that trained athletes exhibited higher cortical excitability post-training or in different exercise states, with increased MEP amplitude, reduced rMT, and shortened MEP latency compared to non-trained individuals or resting states.

**Table 1:** Basic characteristics of the included studies.

Study	Design	Sample (F-female, M-male)	Age E/C (year)	Athlete	Training duration and frequency	Stimulated area	Equipment and parameters	TMS index	Outcome
Fulton et al. (2002)	Pre-post design	11 (all M)	22.37 ± 0.32/22.18 ± 0.26	Rowing	Single session, light intensity for 10 min, high intensity for 1 min	Vertex, targeting bilateral lumbosacral muscles	Single-pulse TMS, double-cone figure-of-eight coil	MEP amplitude	Elite rowers showed less MEP suppression after light training compared to non-athletes.
Perciavalle et al. (2010)	Pre-post design	41 (20F21M)	21.0 ± 1.62/21.7 ± 1.84	Middle- and long-distance runners	3-min no-load warm-up, followed by 30 W increments every 3 min until volitional exhaustion	M1, targeting FDI	Single-pulse TMS, 9 cm circular coil	MT	Enhanced M1 excitability in athletes and a rise in blood lactate levels.
Moscatelli et al. (2016a)	RCT	26 (all M)	25.0 ± 5.0/26.7 ± 6.2	Karate	At least 10 years, 5 times/week, 2 h/session	Left M1, targeting right FDI	Single-pulse TMS, 70 mm figure-of-eight coil	RMT	Karate athletes exhibited higher corticospinal excitability compared to non-athletes.
Moscatelli et al. (2016b)	RCT	50 (all M)	24.9 ± 4.9/26.2 ± 4.5	Karate	Long-term:≥5 years, 5 times/week, 2 h/session	Left M1, targeting right FDI	Single-pulse TMS, 70 mm figure-of-eight coil	RMT	Karate athletes exhibited higher corticospinal excitability compared to non-athletes.
Moscatelli et al. (2016c)	Pre-post design	24 (all M)	24.9 ± 4.9 /26.2 ± 4.5	Taekwondo	Long-term:≥5 years, 5 times/week, 2 h/session	Left M1, targeting right FDS	Single-pulse TMS, 70 mm figure-of-eight coil	RMT	Blood lactate had a greater impact on taekwondo athletes compared to untrained.
Monda et al. (2017)	Cross-sectional study	50 (all M)	24 ± 4	Karate	Weekly 5 times, 2 h/session, ≥5 years	Left M1, targeting FDI	Single-pulse TMS, 70 mm figure-of-eight coil	RMT	Karate athletes showed higher cortical excitability compared to untrained.
Christiansen et al. (2017)	RCT	23 (all M)	25.0 ± 5.0 /26.7 ± 6.2	Non-professional athletes (visuomotor tracking task)	6 weeks, 18 sessions (3 times/week, 7 × 4-min sessions with 2-min rest intervals)	The ipsilateral M1 of the left ADM	Single-pulse TMS, 90 mm figure-eight coil	RMT	Athletes exhibited higher cortical excitability compared to untrained.
Moscatelli et al. (2023)	Matched trial design	20 (all F)	26.5 ± 5.0/25.5 ± 4.8	Professional volleyball	Long-term volleyball training (5 times/week, 2 h/session, ≥5 years)	DLPFC, targeting right FDI	Single-pulse TMS, 80 mm figure-eight coil	RMT	The cortical excitability of volleyball players is enhanced, manifested as a decrease in RMT.

ADM, abductor digiti minimi; DLPFC, Dorsolateral Prefrontal Cortex; FDI, First Dorsal Interosseous; FDS, flexor digitorum superficialis; HF-rTMS, High-Frequency Repetitive Transcranial Magnetic Stimulation; RMT, Resting Motor Threshold; MT, Motor Threshold; MEP, Motor-Evoked Potential; M1, Primary Motor Cortex.

### 3.3 Quality assessment of included studies

**Figure 2:**
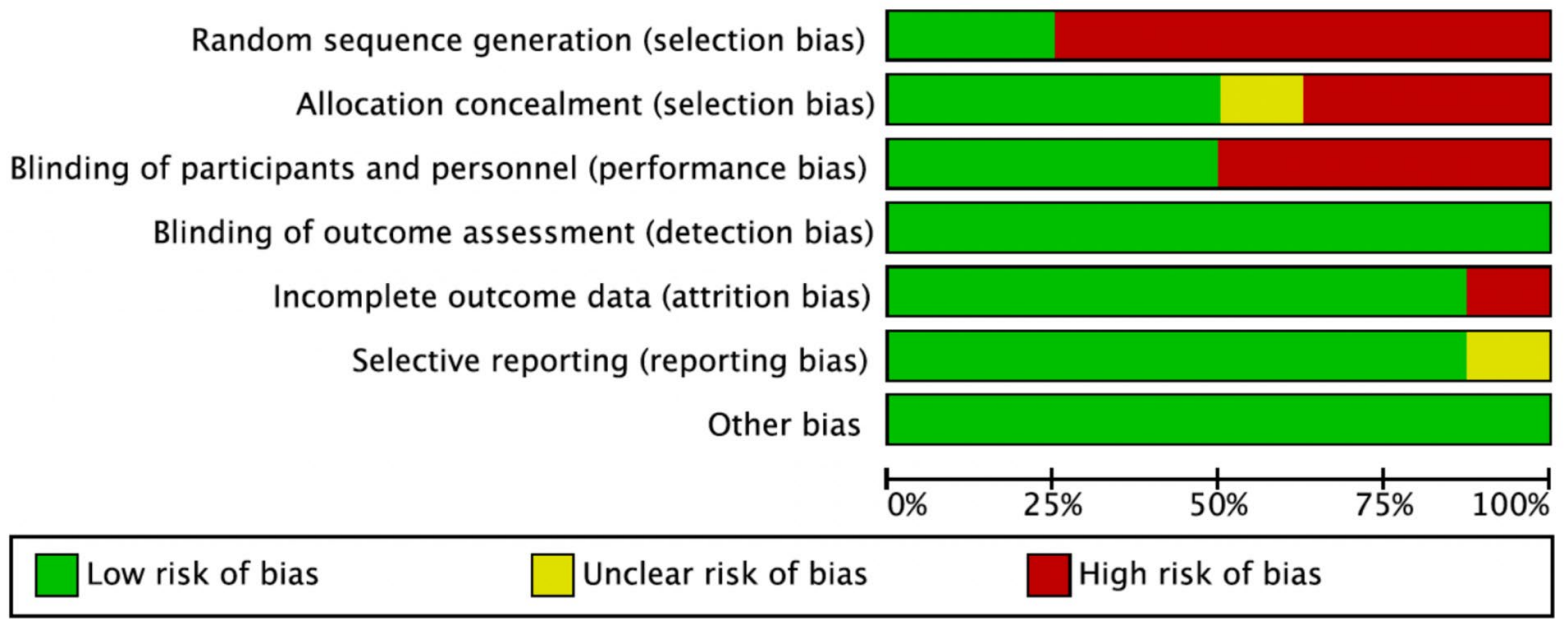
Risk of bias graph.

**Figure 3:**
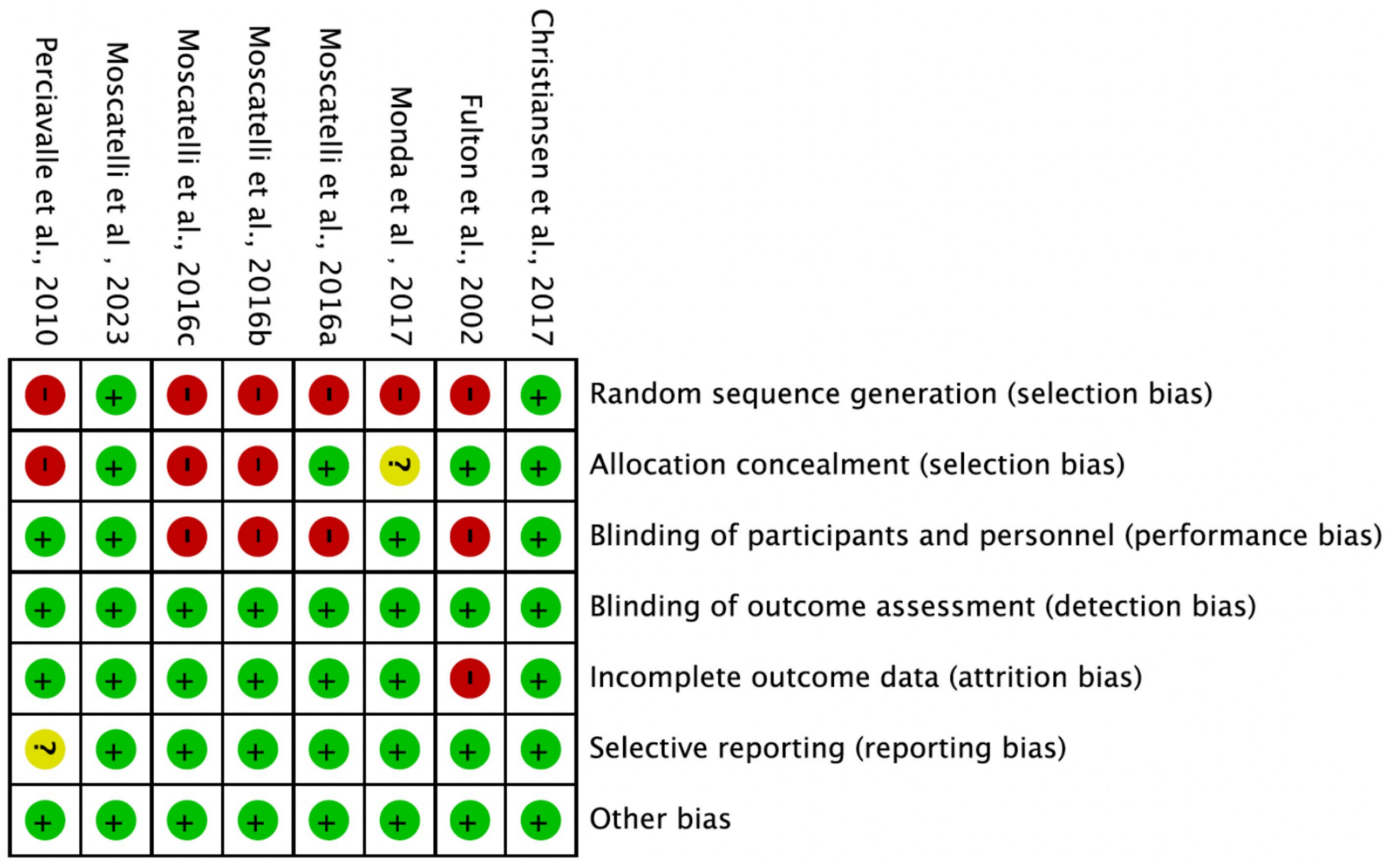
Risk of bias summary.

Among the 8 included studies, the average quality score was 5, with 2 studies reaching 7. As shown in Figure 2, each bar represents a type of bias, with colors indicating risk levels: green (low risk), yellow (unclear risk), and red (high risk). The figure indicates that most studies exhibited low risk across bias categories, though a notable proportion had high or unclear risk in randomization methods and blinding of researchers. Figure 3 details the specific assessment of each bias category for the included studies. Overall, the quality of the included studies was high.

### 3.4 Meta-analysis results

#### 3.4.1 Overall analysis

**Figure 4:**
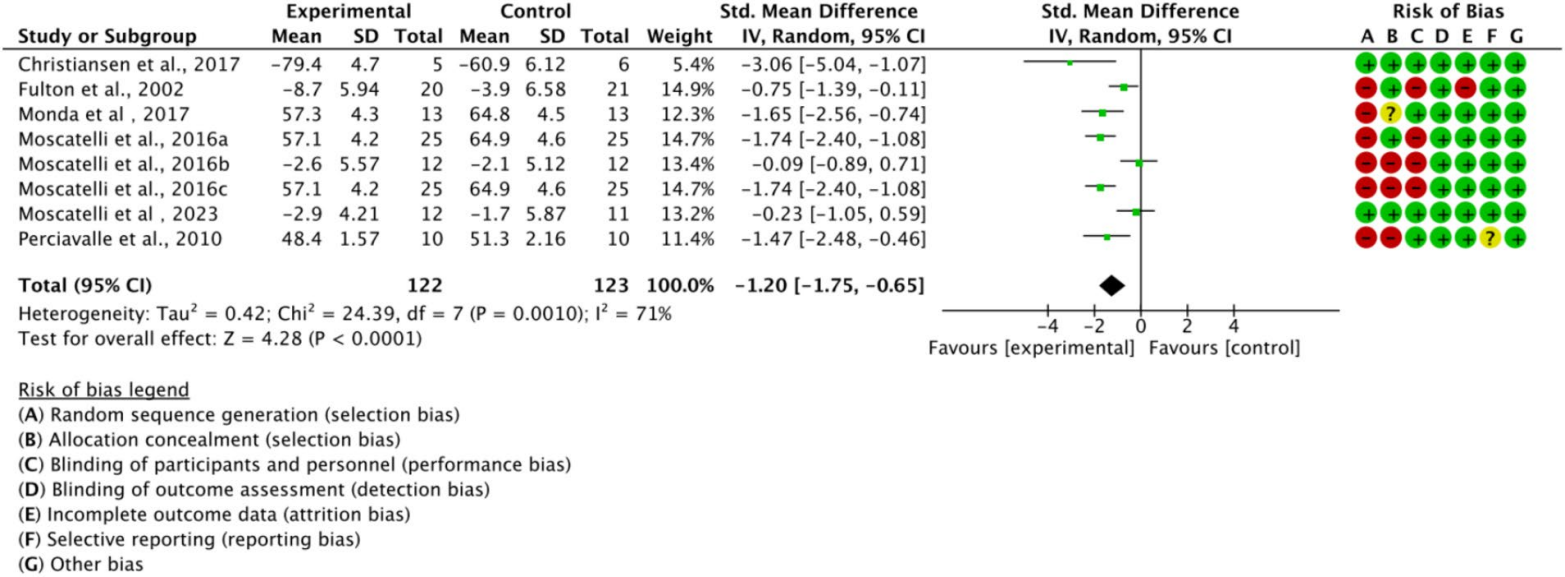
Forest plot of the overall effect of exercise training on cortical excitability in athletes.

Across all included studies, a total of 122 experimental group and 123 control group participants were involved, evaluating the overall effect of exercise training on athletes’ cortical excitability. The forest plot (Figure 4) shows a significant overall effect of exercise training on enhancing cortical excitability *(n = 8, SMD = −1.2, 95% CI = −1.75 to −1.65, p < 0.01)*, indicating that the experimental group exhibited significantly increased cortical excitability compared to the control group, potentially contributing to improved athletic performance. However, high heterogeneity *(I^2^ = 71%)* suggests substantial variability among studies, possibly due to differences in intervention types, sample characteristics, or measurement methods. Additionally, the presence of bias risk in most studies warrants cautious interpretation of results, with further subgroup analysis needed to identify sources of variability.

#### 3.4.2 Subgroup analysis

**Figure 5:**
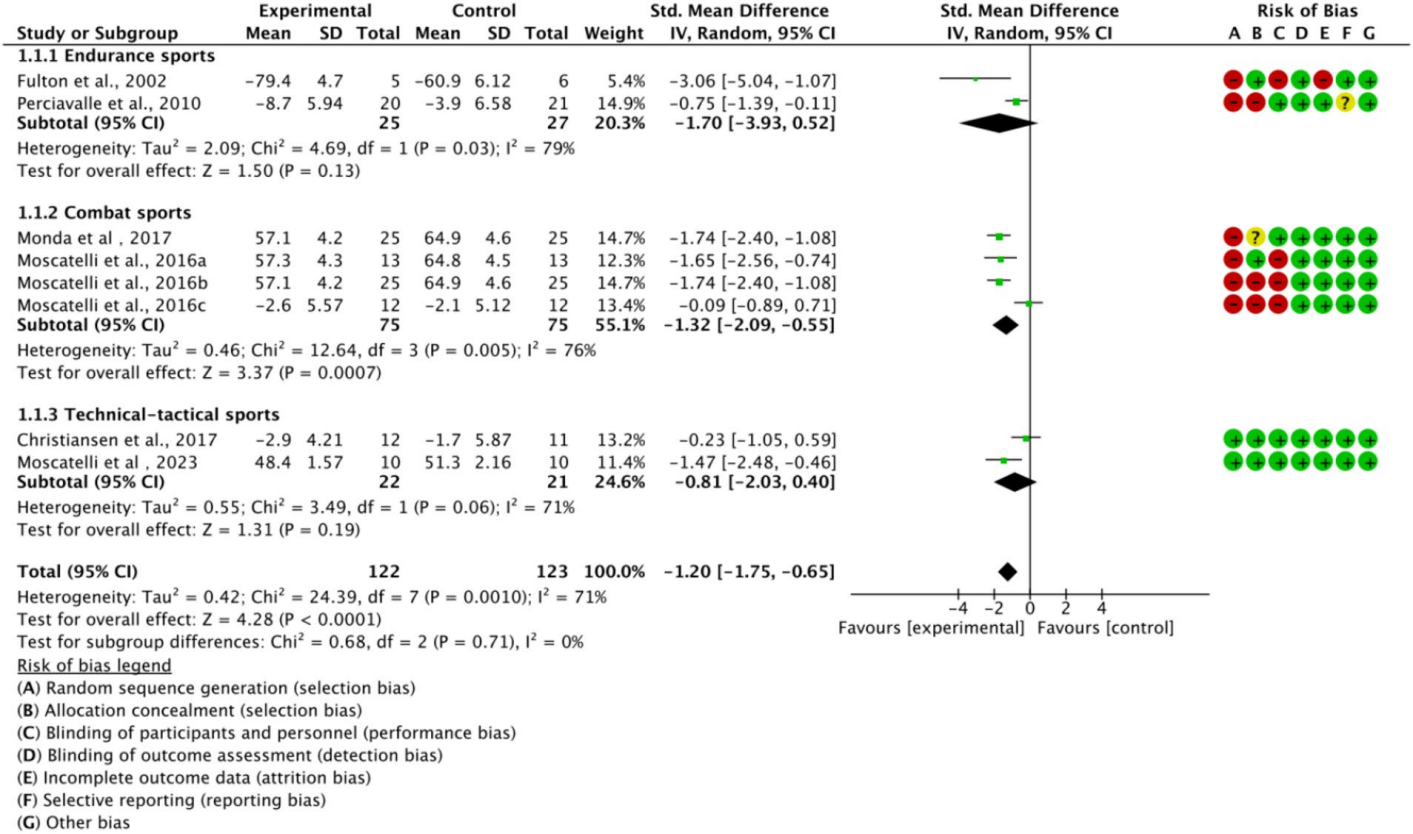
Subgroup analysis of the effect of exercise training on cortical excitability by sport type.

Based on the main movement characteristics and differences in neurophysiological requirements of the various types of movements, they were divided into three subgroups (Figure 5). In the endurance sports subgroup *(n = 2, SMD = −1.70, 95% CI = −3.93 to 0.52, p < 0.05)* and the combat sports subgroup *(n = 4, SMD = −1.32, 95% CI = −2.09 to −0.55, p < 0.05)*, the experimental group showed a trend of difference compared to the control group, with statistically significant results. However, in the technical-tactical sports subgroup, a moderate effect trend was observed *(n = 2, SMD = −0.81, 95% CI = −2.03 to 0.41, p = 0.06)*, but the difference did not reach statistical significance. Additionally, no significant differences were observed between subgroups *(p = 0.71)*.

**Figure 6:**
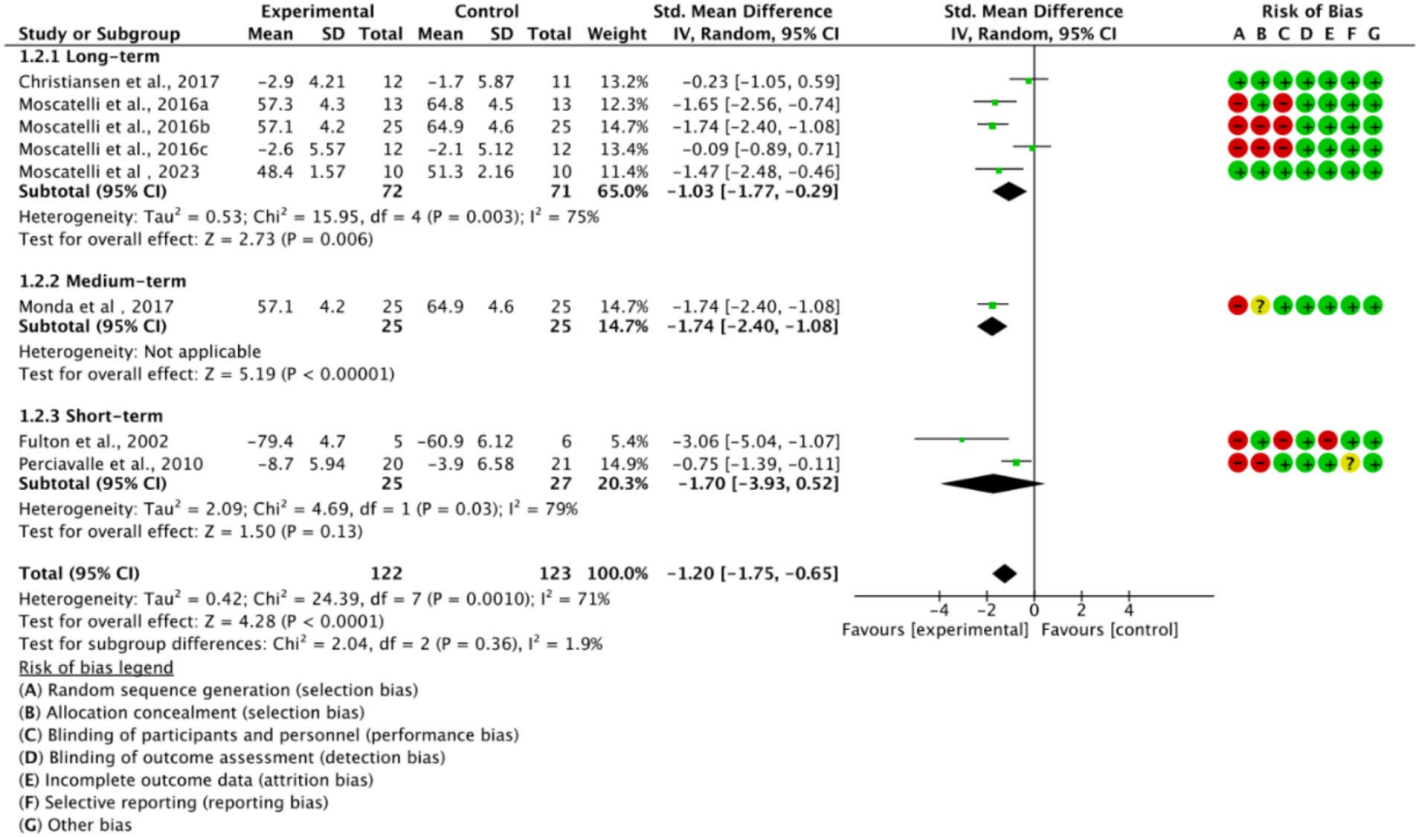
Subgroup analysis based on training duration.

Based on training duration, the included studies were divided into long-term, medium-term, and short-term training subgroups (Figure 6). All three subgroups showed significant effects on cortical excitability *(p < 0.05)*. The medium-term training subgroup exhibited low heterogeneity *(I^2^ = 0%)*, while long-term and short-term training subgroups displayed higher heterogeneity *(I^2^ > 50%)*. This suggests that high heterogeneity is related to training duration.

### 3.5 Publication bias analysis

**Figure 7:**
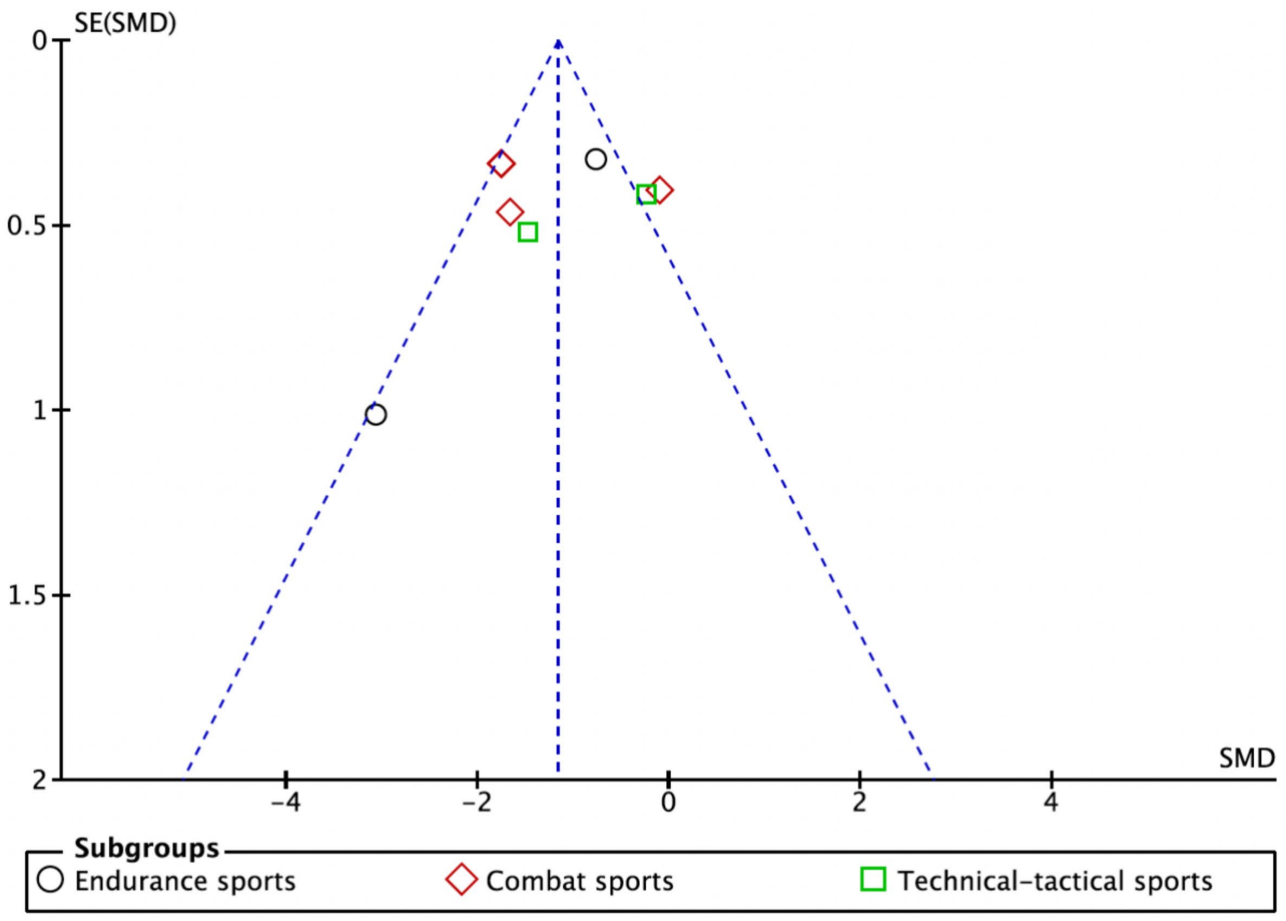
Funnel plot.

As illustrated in Figure 7, data points are represented by circles for the endurance subgroup, diamonds for the combat subgroup, and squares for the technical-tactical subgroup. The distribution of data points across the funnel plot shows variability, with some points clustering near the blue dashed line. Despite this dispersion, there is no evident clustering bias, suggesting a relatively balanced distribution of data across subgroups and no significant distributional anomalies attributable to subgroup differences. Although a few studies appear at the edges of the funnel plot, potentially indicating small-sample effects, the overall plot is largely symmetrical, indicating a low risk of publication bias in this meta-analysis.

**Figure 8:**
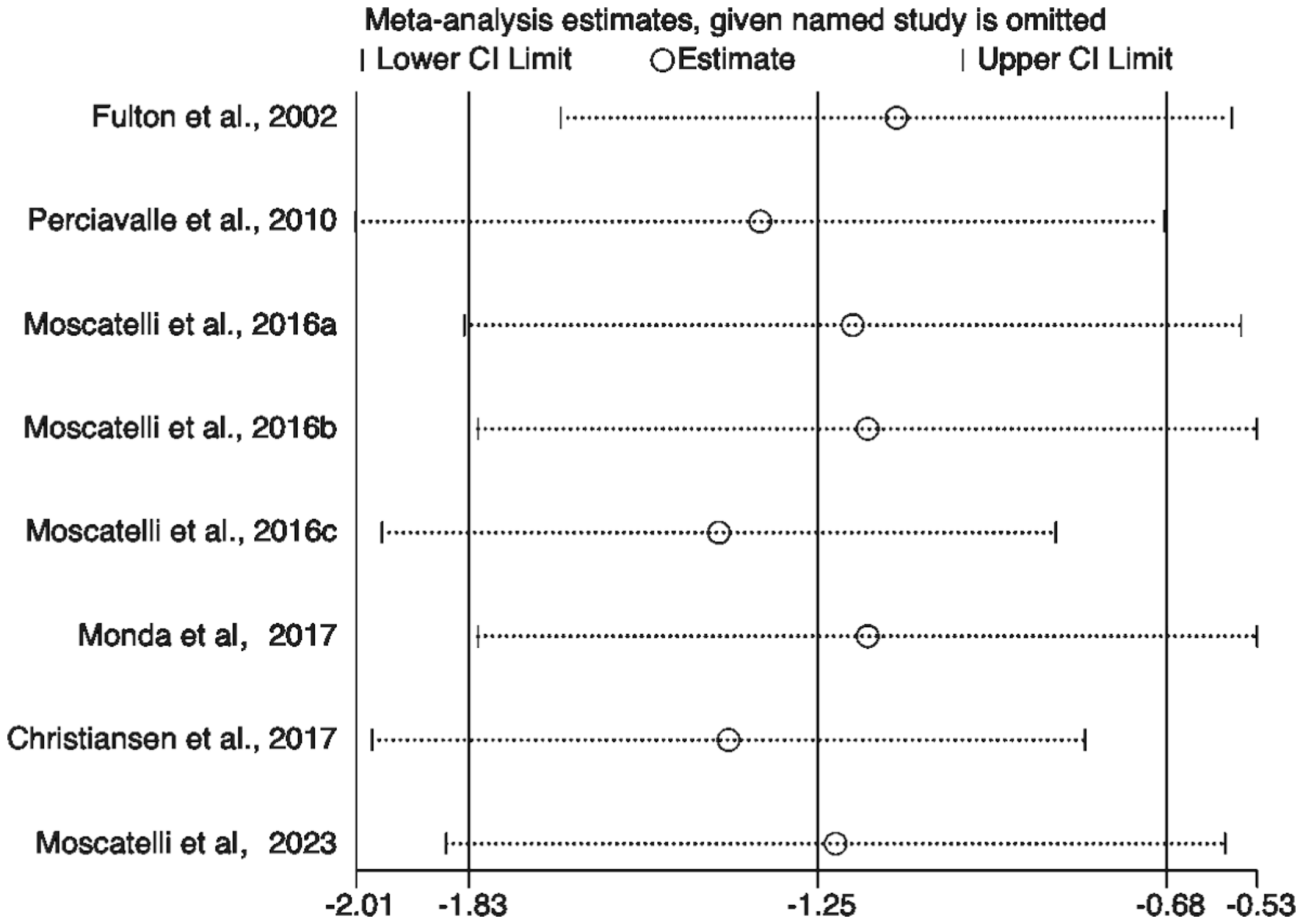
Sensitivity analysis of included studies.

**Figure 9:**
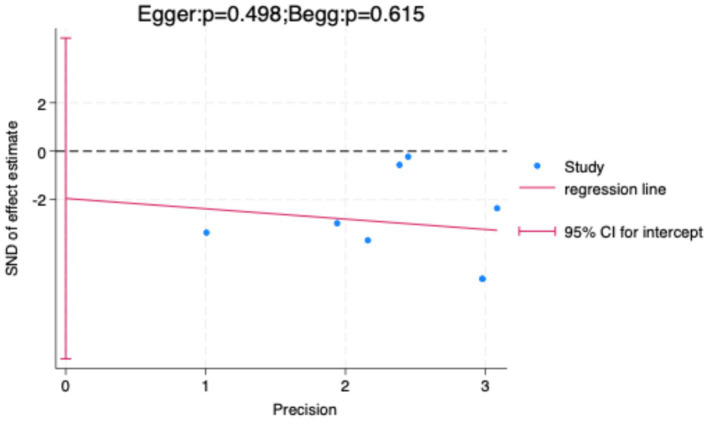
Publication bias assessment using Egger’s and Begg’s tests.

To further validate the findings, sensitivity analysis results (Figure 8) showed that sequentially excluding each included study did not significantly alter the pooled effect size or its confidence interval, indicating strong robustness of the meta-analysis results, independent of any single study. Additionally, both the Egger test and Begg test yielded *p*-values greater than 0.05 (Figure 9), suggesting no significant publication bias.

## 4 Discussion

Exercise training significantly enhances cortical excitability, consistent with findings by Moscatelli et al., who reported increased cortical excitability in karate and taekwondo athletes, suggesting that long-term high-intensity training may promote neural plasticity through repeated activation of motor circuits and strengthened synaptic connections (Moscatelli et al., 2016a; Moscatelli et al., 2016b; Moscatelli et al., 2016c). However, Spampinato et al. (2023) noted that MEP amplitude is influenced by multiple neural pathways, including cortical, spinal, and peripheral levels, indicating that enhanced cortical excitability may not solely result from cortical changes but could also involve alterations in spinal motor neuron or peripheral motor unit synchrony. Additionally, sport-specific characteristics, such as training intensity and skill acquisition patterns, may differentially modulate cortical plasticity.

### 4.1 Interpretation of subgroup analysis

Subgroup analysis by sport type revealed that combat and endurance sports significantly enhanced cortical excitability (*p* < 0.05), while technical-tactical sports did not reach statistical significance (*p* = 0.06). The significant effect in combat sports may be attributed to their high-intensity, explosive characteristics, which emphasize maximal motor unit recruitment, potentially enhancing cortico-spinal pathway efficiency through mechanisms resembling long-term potentiation (LTP) (Ibáñez et al., 2020; Rioult-Pedotti et al., 2000). Studies suggest that high-intensity interval training, such as antagonistic training in combat sports, can induce synaptic plasticity in cortical neurons, enhancing glutamate-mediated excitatory synaptic transmission, thereby reducing resting motor threshold (rMT) and increasing motor evoked potential (MEP) amplitude (Adkins et al., 2006; Coco et al., 2016). For instance, Ibáñez et al. found through TMS that reaction time tasks significantly enhanced intracortical excitatory circuits, particularly with increased I-wave recruitment efficiency in the primary motor cortex (M1), aligning with the high neural efficiency observed in combat athletes (Ibáñez et al., 2020). Additionally, combat sports often involve complex sensory-motor integration and rapid decision-making, which may further stimulate synergistic interactions between the dorsolateral prefrontal cortex (DLPFC) and M1, amplifying cortical excitability (Pascual-Leone et al., 1995).

In endurance sports, training emphasizes sustained aerobic load and rhythmic movements, with cortical plasticity promoted primarily through metabolic and neurochemical mechanisms (Höllge et al., 1997b) (Zghal et al., 2014). Long-term endurance training increases cerebral blood flow and oxygenation, enhances glutamatergic transmission efficiency, and upregulates neurotrophic factors such as BDNF, providing a neurobiological basis for enhanced cortical excitability. In contrast, technical-tactical sports focus on precision and coordination, likely relying more on sensory-motor integration and cortico-cortical network coordination rather than solely on cortico-spinal excitability enhancement (Allen et al., 2007). Karlinsky et al., using TMS combined with functional magnetic resonance imaging (fMRI), found that technical-tactical sports predominantly activate the supplementary motor area (SMA) and premotor cortex (PMC), with relatively smaller excitability changes in M1, which may explain the weaker effect in this subgroup (Karlinsky et al., 2017). Furthermore, the typically lower training intensity in technical-tactical sports compared to combat sports may contribute to limited excitability enhancements due to differences in neural demands (Pearce et al., 2013).

Subgroup analysis by training duration showed that long-term, medium-term, and short-term training all significantly enhanced cortical excitability *(p < 0.05),* indicating that regular exercise interventions, regardless of duration, have the potential to induce cortical plasticity changes. However, differences in effect consistency were observed across training durations. The medium-term training group exhibited the lowest heterogeneity *(I^2^ = 0%)*, suggesting that training protocols in this phase were relatively consistent in design, intensity control, and neural adaptation patterns, yielding stable and reproducible results. In contrast, short-term training interventions, due to their shorter duration, showed greater variability in results, likely due to differences in single-session training load, frequency, and baseline participant conditions, leading to higher heterogeneity. Over longer durations, differences in cumulative training volume, load progression, participant compliance, and effects of fatigue or overtraining further amplified result inconsistency.

### 4.2 Discussion of neurobiological mechanisms

From a neurobiological perspective, exercise-induced cortical adaptations involve not only synaptic-level long-term potentiation (LTP) and long-term depression (LTD) but also the synergistic action of multiple cellular and molecular mechanisms (Cotman et al., 2007; Voss et al., 2013). Firstly, the glutamatergic system, through NMDA receptor-mediated calcium influx, plays a critical role in LTP formation, while the GABAergic system regulates inhibitory synaptic transmission to maintain the excitatory-inhibitory balance, collectively determining the plasticity range of cortical excitability (Lista and Sorrentino, 2010). Secondly, exercise training is associated with structural plasticity changes, such as increased dendritic spine density, synaptic remodeling, and enhanced white matter tract connectivity, providing an anatomical basis for functional adaptations (Pedersen, 2019). Additionally, exercise significantly upregulates neurotrophic factors like BDNF, IGF-1 and VEGF, which further enhance cortical adaptability by promoting synaptic plasticity, neuronal survival, and angiogenesis. In summary, exercise-induced enhancement of cortical excitability should be understood as a multilevel, multi-mechanism process encompassing neurotransmitter regulation, synaptic and network remodeling, and neurotrophic factor support, providing a crucial biological basis for explaining the differential effects of various training modalities on cortical plasticity.

### 4.3 Limitations and recommendations for improvement

This study has several limitations that may affect the interpretation of the results. First, most of the studies followed the basic safety guidelines, but lacked detailed reports on aspects such as coil positioning, stimulus intensity calibration, and control of confounding factors. As a result, they deviated from the standardized methods recommended by IFCN (Groppa et al., 2012; Rossi et al., 2021; Seeck et al., 2017). Second, the methodological quality of the studies was moderate, and some studies failed to adequately control for confounding variables known to influence cortical excitability, such as circadian rhythms, caffeine intake, physical activity prior to TMS measurement, and muscle activation state. These factors may weaken the internal validity of the results and contribute to inter-study heterogeneity. Additionally, the included studies generally lacked standardized data collection and analysis protocols. In recent years, the TMS research field has increasingly emphasized the importance of methodological consistency, proposing standardized frameworks and toolkits to improve study quality and reproducibility. For example, the Brain Electrophysiology Recording and Stimulation (BEST) toolbox (Hassan et al., 2022) provides a systematic approach for TMS data collection, processing, and analysis, while the TMS-EEG guidelines (Rogasch et al., 2017) and international consensus recommendations for TMS methods (Rossi et al., 2021) offer standardized protocols for study design and reporting. These frameworks have been shown to reduce inter-study heterogeneity and enhance cross-study comparability. Future TMS studies in sports and exercise science should prioritize adopting these validated protocols and tools to promote methodological consistency, enhance result reproducibility, and provide a more robust data foundation for subsequent meta-analyses. Finally, this study did not include cardiovascular metrics (e.g., VO₂ max, HRV) or their relationships with cortical excitability and athletic performance. Due to the strict systematic search and inclusion criteria limited to studies using TMS to measure cortical excitability, existing literature did not concurrently report these metrics. The absence of such data limits the ability to comprehensively interpret the role of TMS in optimizing athletic performance from a multidimensional perspective. Future research should design comprehensive studies integrating neurophysiological measures, cardiovascular metrics, and sport-specific performance indicators to develop more predictive and applicable models.

Although the study primarily relied on standardized mean difference (SMD) to pool effect sizes, interpreting results solely based on statistical significance may overlook their clinical and practical importance. Research (Kiers, 1997; Okamoto et al., 2015) indicates that MEP amplitude changes of approximately 20–30% are typically considered neurophysiologic ally significant, reflecting genuine enhancements in cortico-spinal pathway excitability, while changes below this range may partly stem from measurement error or individual state fluctuations. Similarly, a reduction in RMT by 5–10% of stimulation intensity is generally interpreted as an indication of enhanced corticospinal excitability, exceeding the range of normal diurnal variation (Biabani et al., 2021). Therefore, the observed effects in this study should not only be evaluated for statistical significance but also interpreted considering these reference thresholds to assess their practical value in motor neural regulation and skill performance enhancement. Future studies should prioritize this dual-interpretation framework to enhance the translational value of neurophysiological findings.

### 4.4 Practical implications

TMS, as a non-invasive neurophysiological tool, shows considerable potential for monitoring training-induced changes in cortical excitability. However, there is currently insufficient evidence to support its use as a standardized measure of performance. Existing research primarily focuses on the relationship between TMS-derived indices and changes in cortical excitability, but enhanced cortical excitability may also be associated with cardiovascular fitness, reaction speed, and athletic performance. On one hand, VO₂ max and heart rate variability (HRV), which are widely used indicators of exercise capacity and recovery status, have attracted attention for their potential connection with neural plasticity. Although the studies included in this analysis did not directly report VO₂ max or HRV, existing literature suggests that enhanced cortical excitability measured by TMS may act synergistically with improvements in these cardiovascular functions. Future research should adopt a multimodal monitoring approach, integrating TMS metrics with VO₂ max, HRV, and sport-specific performance indicators to develop comprehensive neuro-physiological-behavioral models, and to explore predictive tools for athletic performance or personalized training optimization based on such models.

## 5 Conclusion

Exercise training significantly enhances cortical excitability in athletes, with particularly pronounced effects in combat sports. This finding provides critical evidence for understanding the impact of exercise training on neural plasticity and underscores the utility of TMS as an effective tool for monitoring neural adaptations. Future research should refine experimental designs to explore the relationships between different training modalities and neural mechanisms, thereby advancing the scientific and personalized development of exercise training.

## Data Availability

The original contributions presented in the study are included in the article/Supplementary material, further inquiries can be directed to the corresponding author/s.
