# Supplementary material for: A study on the effects of exercise training on cortical excitability in athletes: a meta-analysis based on TMS measurements
Source: Front Psychol. 2025 Sep 19;16:1627227. doi: 10.3389/fpsyg.2025.1627227 (PMC12491180; doi:10.3389/fpsyg.2025.1627227)
Supplement: Supplementary file 1 [file Supplementary_file_1.docx]

**Supplementary 1. The exact retrieval strategies**

**Pubmed（301）**

(("Athletes"[Mesh] OR athlete* OR sport* OR player*) AND ("Exercise"[Mesh] OR "Exercise Therapy"[Mesh] OR training OR "physical activity" OR "resistance training" OR "aerobic training") AND ("Transcranial Magnetic Stimulation"[Mesh] OR TMS OR "repetitive transcranial magnetic stimulation" OR rTMS) AND ("Cortical Excitability"[Mesh] OR "Motor Cortex Excitability" OR corticospinal excitability))

**WOS（382）**

TS = ((exercise OR training OR "physical activity" OR "resistance training" OR "aerobic training") AND ("transcranial magnetic stimulation" OR TMS OR rTMS) AND ("cortical excitability" OR "motor cortex" OR "corticospinal" OR neuroplasticity) AND (athlete* OR sport* OR player* OR volunteer*))

**Cochrane Library（111）**

(athlete* OR sport* OR player* OR "healthy subject*" OR volunteer*) AND (exercise OR training OR "physical activity" OR "resistance training" OR "aerobic training") AND ("transcranial magnetic stimulation" OR TMS OR rTMS) AND ("cortical excitability" OR "motor cortex" OR corticospinal OR neuroplasticity)ty" OR arm OR hand) AND ("motor function" OR "motor recovery" OR "motor impairment")

**Embase（175）**

('athlete'/exp OR athlete* OR sport* OR player*) AND ('exercise'/exp OR 'exercise therapy'/exp OR training OR "physical activity" OR "resistance training" OR "aerobic training") AND ('transcranial magnetic stimulation'/exp OR TMS OR rTMS) AND ('cortical excitability'/exp OR "motor cortex excitability" OR "corticospinal excitability")

**Supplementary 2 Meta-regression**

**
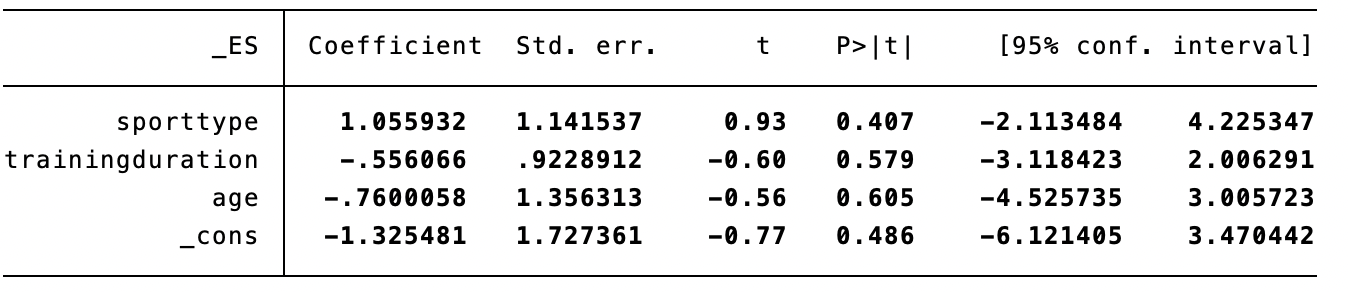
**
